# Sparse Graph Regularization Non-Negative Matrix Factorization Based on Huber Loss Model for Cancer Data Analysis

**DOI:** 10.3389/fgene.2019.01054

**Published:** 2019-11-20

**Authors:** Chuan-Yuan Wang, Jin-Xing Liu, Na Yu, Chun-Hou Zheng

**Affiliations:** ^1^ School of Information Science and Engineering, Qufu Normal University, Rizhao, China; ^2^ School of Software Engineering, Qufu Normal University, Qufu, China

**Keywords:** non-negative matrix factorization, Huber loss, sample clustering, graph regularization, robustness

## Abstract

Non-negative matrix factorization (NMF) is a matrix decomposition method based on the square loss function. To exploit cancer information, cancer gene expression data often uses the NMF method to reduce dimensionality. Gene expression data usually have some noise and outliers, while the original NMF loss function is very sensitive to non-Gaussian noise. To improve the robustness and clustering performance of the algorithm, we propose a sparse graph regularization NMF based on Huber loss model for cancer data analysis (Huber-SGNMF). Huber loss is a function between *L*_1_-norm and *L*_2_-norm that can effectively handle non-Gaussian noise and outliers. Taking into account the sparsity matrix and data geometry information, sparse penalty and graph regularization terms are introduced into the model to enhance matrix sparsity and capture data manifold structure. Before the experiment, we first analyzed the robustness of Huber-SGNMF and other models. Experiments on The Cancer Genome Atlas (TCGA) data have shown that Huber-SGNMF performs better than other most advanced methods in sample clustering and differentially expressed gene selection.

## Introduction

Cancer is considered to be the number one killer of human health. The development of high-throughput sequencing technology has enabled researchers to obtain more comprehensive information about cancer patients (Chen et al., 2019). The gene expression data of cancer patients can be more used for effective data mining through computational methods (Chen et al., 2018). In general, cancer gene expression data are characterized by high dimensionality, which is extremely difficult for data analysis. How to effectively reduce the dimensionality of data is the key to analyzing cancer data. Principal component analysis (PCA) (Feng et al., 2019), locally linear embedding (LLE) (Roweis and Saul, 2000), and non-negative matrix factorization (NMF) (Yu et al., 2017) are common methods for reducing the data dimensionality. Unlike several other methods, NMF can find two non-negative matrices and its product can effectively restore the original matrix. The non-negative constraint guarantees additive combinations between different elements. NMF demonstrates its advantages in facial recognition, speech processing, document clustering, and recommendation systems (Guillamet and Vitrià, 2002; Xu et al., 2003; Schmidt and Olsson, 2006; Luo et al., 2014).

NMF has developed rapidly in recent years, and several variants of NMF have been proposed to improve the effectiveness of the decomposition. Cai et al. proposed graph regularized NMF (GNMF) for data representation (Cai et al., 2011). GNMF considers the association between points to preserve the internal structure of the data. Kim et al. applied the *L* ^1^-norm constraint on the coefficient matrix to introduce sparse NMF for clustering (SNMF) (Kim and Park, 2007). Sparseness is more likely to remove redundant features of data. The most of cancer data have noise and outliers, and the original NMF cannot solve this. Wang et al. introduced Characteristic Gene Selection Based on Robust GNMF (RGNMF) (Wang et al., 2016a) to improve the robustness of the algorithm. RGNMF assumes that the loss follows Laplacian distribution and uses the loss function of the *L* ^2,1^-norm (Kong et al., 2011) constraint. The *L* ^2,1^-norm combines the advantages of the *L* ^2^-norm and the *L* ^1^-norm, which impose an *L* ^2^-norm constraint on the entire data space and an *L* ^1^-norm constraint on the sum of the different data points (Ding et al., 2006).

The original NMF model is simple to understand and computationally fast, but the squared loss function is too sensitive to outliers and noise. Mao et al. proposed the correntropy induced metric based GNMF (CGNMF) (Mao et al., 2014) that changed the original loss function. The correntropy uses *L* ^0^-norm approximation for large outliers and noise through kernel function filtering, and the normal data is constrained by the *L* ^2^-norm (Liu et al., 2007), so it is not sensitive to outliers and noise. Du et al. proposed Huber-NMF (Du et al., 2012), which is also a loss function that is insensitive to outliers and noise. It uses approximate *L* ^1^-norm processing for outliers and noise, and *L* ^2^-norm for valuable data. Correntropy uses kernel functions to control weights, and Huber loss uses a different function approximation for different data through threshold adjustment. The robustness analysis of these several non-square loss models is given in the experimental part. To compare the performance of the NMF algorithm, the robust PCA (RPCA) based method for discovering differentially expressed genes proposed by Liu et al. (2013) is added to the experiment.

In this paper, we propose a model called sparse graph regularization NMF based on Huber Loss Model for Cancer Data Analysis (Huber-SGNMF). It effectively combines Huber loss, manifold structure, and sparse constraint. Huber loss is based on the relationship between *L* ^1^-norm and *L* ^2^-norm to approximate different data. In detail, Huber loss adjusts the square loss or linear loss to the data according to the threshold to enhance the robustness of the model to outliers. Geometric information in high-dimensional data should remain locally constant in low-dimensional representations (Cai et al., 2011), so graph regularization is added to preserve the manifold structure of the data. Sparse constraints in the model can remove redundant features contained in the data to reduce the amount of model calculations and improve clustering performance (Kim and Park, 2007).

The contributions of this article are as follows:

1. The squared loss of the original NMF is too sensitive to outliers and noise; so, we use a more robust Huber loss combined with NMF. The Huber loss considers the relationship between the *L* ^1^-norm and the *L* ^2^-norm to effectively handle non-Gaussian noise and large outliers. For the update rules of Huber loss, we use the multiplicative iterative algorithm based on semi-quadratic optimization to find the optimal solution.
2. The NMF model fits the data in Euclidean space but does not consider the intrinsic geometry of the data space. If the data is related in high-dimensional space, then we believe that the data represented by the low-dimensional should also be closely related. Considering the manifolds embedded in the high-dimensional environment space, we add graph Laplacian as a regularization term to the model. Graph regularization takes into account the impact of recent neighbors on data, and retaining graph structure can make NMF more distinguishable.
3. Sparse matrices can remove redundant data, reducing data complexity and model computational difficulty. In data analysis, sparsity can improve clustering performance by reducing the difficulty of feature selection. The *L* ^2,1^-norm as a sparse constraint is added to the model because the *L* ^2,1^-norm is robust and can achieve row sparse effect.

The remainder of this paper is organized as follows. The introduction of related work is shown in Section 2. Models and solution optimization are presented in Section 3. The experiment and analysis are arranged in Section 4. Section 5 summarizes the entire paper.

## Related Work

### Non-Negative Matrix Factorization

NMF is a dimensionality reduction method based on partial representation. For a given dataset $X=\left[x_{1},x_{2}\ldots ,x_{n}\right]\in {\mathbb{R}}^{m\times n}$, NMF can decompose it into the basic matrix $U\in {\mathbb{R}}^{m\times k}$ and the coefficient matrix $V\in {\mathbb{R}}^{k\times n}$, with the purpose of approximating the original matrix by two matrix products. In general, the rank of matrix factorization *k* is selected by the number of larger singular values.

**Figure 1:**
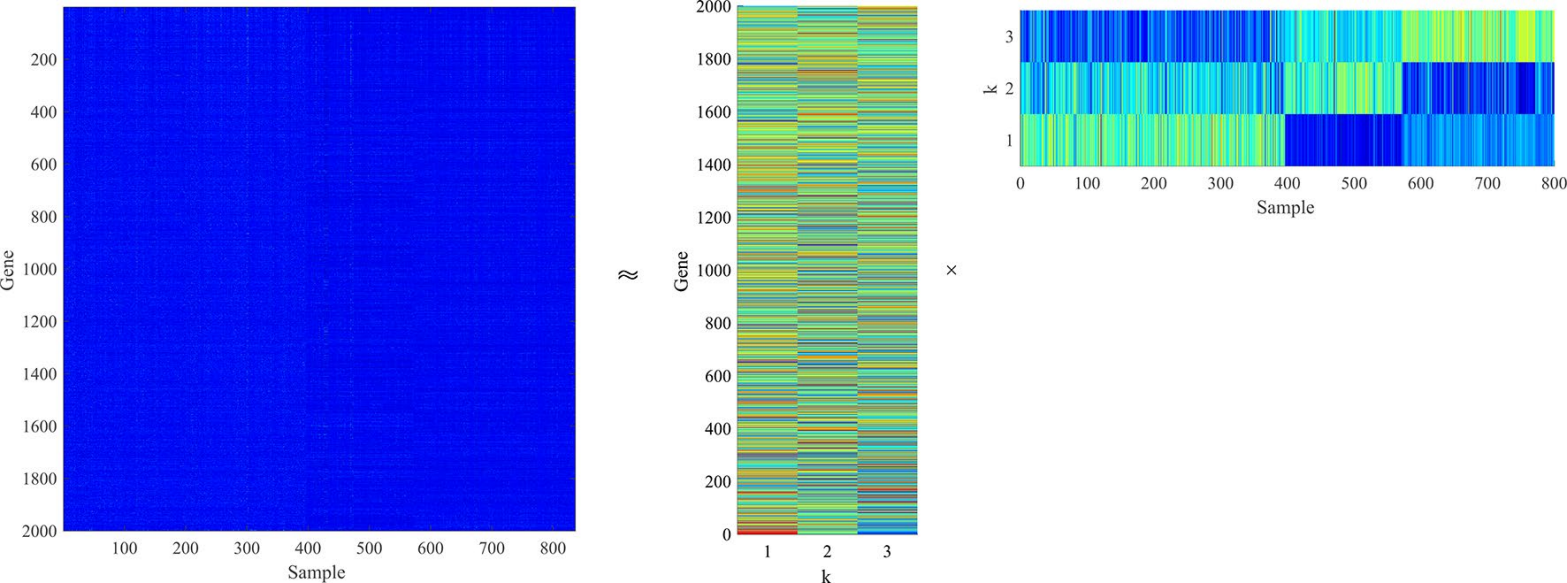
The gene expression data matrix $X\in {\mathbb{R}}^{m\times n}$ is decomposed into a low-dimensional basic matrix $U\in {\mathbb{R}}^{m\times k}$ and a low-dimensional coefficient matrix $V\in {\mathbb{R}}^{k\times n}$. The product of two low-dimensional matrices can approximate the original matrix.

For gene expression data matrix $X\in {\mathbb{R}}^{m\times n}$, each row represents a gene corresponding to *n* samples, and each column represents a sample composed of *m* genes. Moreover, **U** contains *m* rows of metagene and **V** contains *n* rows of metapattern (Liu et al., 2018). Each column of **V** is a projection of a corresponding sample vector in **X** according to the basic matrix **U** (Li et al., 2017). NMF is visualized on gene expression data as shown in **Figure 1**.

The NMF loss function is minimized as follows:

(1) $$\min\;{\left\|X-UV\right\|}^{2},\;s.t.\;U\geq 0,V\geq 0,$$

where ‖⋅‖ represents the application of the Frobenius norm to the matrix.

Lee and Seung proposed the use of multiplicative iterative update rules to solve the optimal solution of NMF (Lee and Seung, 1999). Its update formula is as follows:

(2) $$u_{ik}=u_{ik}\frac{{\left(XV\right)}_{ik}}{{\left(UVV^{T}\right)}_{ik}},$$

(3) $$v_{kj}=v_{kj}\frac{{\left(U^{T}X\right)}_{kj}}{{\left(U^{T}UV\right)}_{kj}},$$

where *u* *_ik_* and *v* *_kj_* are elements belonging to **U** and **V**, respectively. The non-negative constraints of **U** and **V** only allow additive combinations between different elements, so NMF can learn part-based representations (Cai et al., 2011).

### Huber Loss

Data usually contain a small amount of outliers and noise, which can have a worse effect on model reconstruction. For noise and outliers in the dataset, Huber loss uses weighted *L*_1_-norm processing because the *L*_1_-norm is robust and can effectively handle outliers and noise (Guofa et al., 2011; Yu et al., 2016). For other valuable data in the dataset, Huber losses still use *L*_2_-norm loss to fit the data. Huber loss function *δ*(*·*) is defined as follows:

(4) $$\delta \left(e\right)=\left\{ \begin{array}{l} e^{2}\;if\;\left|e\right|<c,\\ 2c\left|e\right|-c^{2}\;if\;\left|e\right|\geq c, \end{array}\right.$$

where *c* represents the threshold parameter of the data using the *L*_1_-norm or the *L*_2_-norm. This function is a bounded and convex function that minimizes the effects of a single anomaly point (Chreiky et al., 2016). Huber losses often apply to the insensitive outliers and noise contained in the data, which are often difficult to find using the squared loss function (Du et al., 2012).

### Manifold Regularization

The manifold learning theory (Belkin and Niyogi, 2001) shows that the internal manifold structure of the data can be effectively simulated by the nearest neighbor of the data points. Each data point finds its nearest *p* neighbors and connects the data points to the neighbors with edges. There are many ways to define the weight of an edge, most commonly 0–1 weighted: **W**_ij_=1, if and only if nodes *i* and *j* are connected by edges. The advantage of this weighting method is that it is easy to calculate.

Weight matrix **W** *_ij_* is only used to measure the intimacy between data points. For the low-dimensional representation **s** *_j_* of the high dimensional data **x** *_j_*, the Euclidean distance $O(s_{j},s_{l})={\left\|s_{j}-s_{l}\right\|}^{2}$ is typically used to measure the similarity between two low-dimensional data points. According to the intimacy weight W, the smoothness of the two low-dimensional vectors can be measured as follows:

(5) R=12∑j,lN‖sj−sl‖2Wjl=∑j=1NsjTsjDjj−∑j,l=1NsjTslWjl=tr(VDVT)−tr(VWVT)=tr(VLVT),

where tr(·) denotes the trace of a matrix. The matrix **D** is defined as a diagonal matrix with diagonal elements $D_{ii}=\sum_{jj}W_{jl}$ The graph Laplacian (Liu et al., 2014) matrix **L** is defined as **L**=**D**-**W**.

We hope that if the high-dimensional data **x** *_j_* and **x** *_l_* are very intimate, then **s** *_j_* and **s** *_l_* should be close enough in low-dimensional representations (Cai et al., 2011). Therefore, minimizing *R* is added to our model to encode the internal geometry of the data.

## Method

### The Huber-Sgnmf Model

Based on the Huber loss function, we proposed a novel model that preserves the manifold structure and sparsity simultaneously. The Huber loss is combined with NMF to enhance NMF robustness. To further optimize the model, the graph regularization term and the *L*_2,1_-norm are added to the loss function as constraints. *L*_2,1_-norm mathematical expression is as follows:

(6) $${\left\|X\right\|}_{2,1}=\sum_{i=1}^{m}\sqrt{\sum_{j=1}^{n}x_{ij}^{2}}=\sum_{i=1}^{m}{\left\|x_{i,*}\right\|}_{2}.$$

The Huber-SGNMF final model *O* *_Huber_*_−_ *_SGNMF_* is as follows:

(7) $$\underset{U\geq 0,V\geq 0}{\min}\;\delta (X-UV)+\alpha \text{ tr}\left(VLV^{T}\right)+\beta {\left\|V\right\|}_{2,1},$$

where tr(·), *α*, and *β* represent the trace of the matrix, the regularization term parameters, and the sparse constraint parameters, respectively. In the experiment, the basic matrix **U** and the coefficient matrix **V** are used for differential gene selection and cluster analysis, respectively.

### Optimization

Obviously, the loss function is a non-quadratic optimization problem, and finding the optimal solution is not simple. Fortunately, the semi-quadratic optimization technique that has been proposed can effectively optimize the loss function. The loss function can be reconstructed to find the optimal solution by introducing auxiliary variables. According to the conjugate function and the semi-quadratic technique (Nikolova and Chan, 2007), the fixed loss function *σ*(**Z**) can be constructed as follows:

(8) $$\sigma \left(Z_{ij}\right)=\underset{W\in \mathbb{R}}{\min}\;\tau \left(Z_{ij},W_{ij}\right)+\phi \left(W_{ij}\right),$$

where $Z_{ij}=X_{ij}-\sum_{k=1}^{K}U_{ik}V_{kj}$ represents the difference between the NMF predicted value and the actual value. *σ*(·) indicates the noise or normal data, which is processed using different loss functions. $W\in {\mathbb{R}}^{m\times n}$ is the introduced auxiliary variable. *ϕ*(**W** *_ij_*) is the conjugate function of **Z** *_ij_*. τ(⋅,⋅) is a quadratic term for **Z** *_ij_* and **W** *_ij_*. The NMF model only needs to consider the quadratic term of the multiplication form:

(9) $$\tau \left(Z_{ij},W_{ij}\right)=\frac{1}{2}W_{ij}Z_{ij}^{2}.$$

Combine Equation (8) and Equation (9) with the loss function (7):

(10) minU≥0,V≥0  δ(X-UV)+αtr(VLVT)+β‖V‖2,1=minU≥0,V≥0  W⊗(X-UV)2+ϕ(W)+αtr(VLVT)+β‖V‖2,1,

where ⊗ represents the Hadamard product, which is the product between two matrices’ elements. Operator ⊗ takes precedence over other matrices operators. Its Lagrangian function expansion is expressed as follows:

(11) $${ℒ}_{Huber-SGNMF}\left(U\right)=\sum_{i=1}^{m}\left(X_{i*}-U_{i*}V_{i*}\right)Q_{i}{\left(X_{i*}-U_{i*}V_{i*}\right)}^{T}+\text{tr}\left(\psi U^{T}\right),$$

and

(12) $$\begin{array}{c} {ℒ}_{Huber-SGNMF}\left(V\right)=\sum_{j=1}^{n}\left(X_{*j}-U_{*j}V_{*j}\right)R_{j}{\left(X_{*j}-U_{*j}V_{*j}\right)}^{T}\\ \;+\alpha \text{tr}\left(VLV^{T}\right)\;+\beta \text{tr}\left(VGV^{T}\right)+tr\left(\psi U^{T}\right)+\text{tr}\left(\varphi V^{T}\right), \end{array}$$

where **Q** *_i_* and **R** *_j_* are defined as **Q** *_i_*=*diag*(**W** _i__*_) and **R**=*diag*(W_*_ *_j_*), respectively. $\psi =\left[\psi_{ik}\right]$ and $\varphi =\left[\varphi_{kj}\right]$ are Lagrangian multipliers of non-negative constraints **U** 0 and **V** 0, respectively. **G** is a diagonal matrix with diagonal elements, which is given by:

(13) $$G_{jj}=1/\sqrt{\sum_{m=1}^{k}v_{mj}+\omega }$$

where *ω* is a number that is very close but not equal to zero.

Let **ψU**=0 and ϕ**V**=0 by using Karush–Kuhn–Tucher (KKT) (Qi and Jiang, 1997) conditions. The loss function (10) can be iteratively optimized by the following schemes:

Update **W** when **U** and **V** are fixed. The weight matrix **W** according to equation (8) is defined as follows:

(14) $$w_{ij}=\frac{\sigma^{\prime }\left(Z_{ij}\right)}{Z_{ij}},$$

where the elements of weight matrix is *w* *_ij_* *ϵ* **W** Combine the loss function (7) with the equation (14) are as follows:

(15) $$w_{ij}=\left\{ \begin{array}{l} \;1\;if\;\left|x_{ij}-u_{ik}v_{kj}\right|\leq c,\\ \frac{c}{{\left|X-UV\right|}_{ij}}\;otherwise, \end{array}\right.$$

Update **U** and **V** when **W** is fixed. The update rules for **U** and **V**are as follows:

(16) $$\begin{array}{l} u_{ik}=u_{ik}\frac{{\left(X_{i*}Q_{i}V^{T}\right)}_{ik}}{{\left(U_{i*}VQ_{i}V^{T}\right)}_{ik}}\\ \;=u_{ik}\frac{{\left(W⊗XV^{T}\right)}_{ik}}{{\left(W⊗\left(UV\right)V^{T}\right)}_{ik}}, \end{array}$$

(17) $$\begin{array}{l} v_{kj}=v_{kj}\frac{{\left(U^{T}X_{*j}R_{j}\right)}_{kj}}{{\left(U^{T}R_{j}UV_{*j}+\alpha VL+\beta VG\right)}_{kj}}\\ \;=v_{kj}\frac{{\left(U^{T}\left(W⊗X\right)\right)}_{kj}}{{\left(U^{T}\left(W⊗UV\right)+\alpha VL+\beta VG\right)}_{kj}}, \end{array}$$

The threshold parameter *c* is set to the median of the reconstruction error,

(18) $$c=\;median\;({\left|X-UV\right|}_{ij}).$$

The corresponding algorithm is shown in Algorithm 1.

**Algorithm 1:** Huber-SGNMF.


Data input: $X\in {\mathbb{R}}^{m\times n},\;L\in {\mathbb{R}}^{n\times n}$
Data output: $U\in {\mathbb{R}}^{m\times k},$ $\;V\in {\mathbb{R}}^{k\times n}$ and weight matrix $\;W\in {\mathbb{R}}^{m\times n}$
Parameters: *α*,*β*
Data initialize: **U**≥0, **V**≥0
Repeat Update **G** by (13); Update **W** by (15);
Update **u** *_ik_* by (16); Update **v** *_jk_* by (17); Update *c* by (18);
End convergence

### Convergence Analysis

According to the update rules of Huber-SGNMF, the loss function *O* *_Huber-SGNMF_* can converge to the local optimum through theorem 1.

**Theorem 1.** The loss function (7) is guaranteed to be non-increasing under the update rules (16) and (17). The loss function is constant when the elements *u* *_ik_* and *v* *_kj_* have fixed values.

To prove theorem 1, we introduce the auxiliary function **H** in Algorithm.

**Lemma 1.** Suppose **H** (*r*, *r*′) is an auxiliary function of F (*r*). If the conditions **H** (*r*,*r*′) F(*r*) and **H** (*r*,*r*)=F(*r*) are satisfied, then it can be concluded that F(*r*) is non-increasing from iteration *t* to *t*+1:

(19) $$r^{t+1}=\arg\;\underset{r}{\min}H(r,r\prime )$$

Proof:

(20) $$\text{F}(r^{t+1})\leq H(r^{t+1},r^{t})\leq H(r^{t},r^{t})=\text{F}(r^{t}).$$

Suppose loss function *O* *_Huber-SGNMF_* has a suitable auxiliary function **H** *_Huber_* If the minimum updates rule for **H** *_Huber_* is equal to (16) and (17), then the convergence of *O* *_Huber-SGNMF_* can be proved. Furthermore, the parts of the loss function *O* *_Huber-SGNMF_* associated with the elements *u* *_ik_* *_ϵ_* **U** and *v* *_kj_* *_ϵ_* **V** are represented by F*_ik_* and F*_kj_*, respectively. The partial derivative equation of *O* *_Huber-SGNMF_* can be derived as follows:

(21) $$F\prime_{ik}={\left(\frac{\partial O_{Huber-SGNMF}}{\partial U}\right)}_{ik}={\left(-2X_{i*}Q_{i}V^{T}+2U_{i*}VQ_{i}V^{T}\right)}_{ik},$$

(22) $$F_{\mathrm{ik}}^{\prime \prime }={\left(\frac{\partial^{2}O_{Huber-SGNMF}}{\partial U^{2}}\right)}_{ik}=2{\left(VQ_{i}V^{T}\right)}_{kk},$$

(23) $$\begin{array}{c} F_{\mathrm{kj}}^{\prime }={\left(\frac{\partial O_{Huber-SGNMF}}{\partial V}\right)}_{kj}=-2{\left(U^{T}X_{*j}R_{j}\right)}_{k}\\ +2{\left(U^{T}R_{j}UV_{*j}\right)}_{k}+2\alpha VL+2\beta VG, \end{array}$$

(24) $$F_{\mathrm{kj}}^{\prime \prime }={\left(\frac{\partial^{2}O_{Huber-SGNMF}}{\partial V^{2}}\right)}_{kj}=2{\left(U^{T}R_{j}U\right)}_{kk}+{\left(2\alpha L\right)}_{jj}+{\left(2\beta G\right)}_{jj}.$$

Essentially, the algorithm updates each element, which means that if the elements F*_ik_* and F*_kj_* are non-increasing, then *O* *_Huber-SGNMF_* is also non-increasing.

**Lemma 2.** Define $H_{Huber}\left(u,u_{ik}^{t}\right)$ and $H_{Huber}\left(v,v_{kj}^{t}\right)$ as auxiliary functions for **u** *_ik_* and **v** *_kj_*, respectively. The expansion items are as follows:

(25) $$\begin{array}{l} H_{Huber}\left(u,u_{ik}^{t}\right)=F_{ik}\left(u_{ik}^{t}\right)+F_{\mathrm{ik}}^{\prime }\left(u_{ik}^{t}\right)\left(u-u_{ik}^{t}\right)\\ \;+\frac{{\left(U_{i*}VQ_{i}V^{T}\right)}_{ik}}{u_{ik}^{t}}{\left(u-u_{ik}^{t}\right)}^{2}, \end{array}$$

(26) $$\begin{array}{l} H_{Huber}\left(v,v_{kj}^{t}\right)=F_{kj}\left(v_{kj}^{t}\right)+F_{\mathrm{kj}}^{\prime }\left(v_{kj}^{t}\right)\left(v-v_{kj}^{t}\right)\\ \;+\frac{{\left(U^{T}R_{j}UV_{*j}+\alpha VL+\beta VG\right)}_{kj}}{v_{kj}^{t}}{\left(v-v_{kj}^{t}\right)}^{2}. \end{array}$$

Proof:

According to the lemma 1, **H** *_Huber_* (*u,u*)*=*F*_ik_*(*u*) and **H** *_Huber_* (*v,v*)=F*_kj_*(*v*) can be obtained. We have the following formulas through the Taylor series expansion of the auxiliary function.

(27) $$\begin{array}{l} {\text{F}}_{ik}\left(u\right)\approx {\text{F}}_{ik}\left(u_{ik}^{t}\right)+{\text{F}}_{\mathrm{ik}}^{\prime }\left(u_{ik}^{t}\right)\left(u-u_{ik}^{t}\right)\\ \;+\frac{1}{2}{\text{F}}_{\mathrm{ik}}^{\prime \prime }\left(u_{ik}^{t}\right){\left(u-u_{ik}^{t}\right)}^{2}, \end{array}$$

(28) $$\begin{array}{l} {\text{F}}_{kj}\left(v\right)\approx {\text{F}}_{kj}\left(v_{kj}^{t}\right)+{\text{F}}_{\mathrm{kj}}^{\prime }\left(v_{kj}^{t}\right)\left(v-v_{kj}^{t}\right)\\ \;+\frac{1}{2}{\text{F}}_{\mathrm{kj}}^{\prime \prime }\left(v_{kj}^{t}\right){\left(v-v_{kj}^{t}\right)}^{2}. \end{array}$$

Next, $H_{Huber}\left(u,u_{ik}^{t}\right)\geq F_{ik}\left(u\right)$ and $H_{Huber}\left(v,v_{kj}^{t}\right)\geq F_{kj}\left(v\right)$ need to be guaranteed.

According to (25) and (27), expand $H_{Huber}\left(u,u_{ik}^{t}\right)\geq F_{ik}\left(u\right)$ is as follows:

(29) $$\frac{{\left(U_{i*}VQ_{i}V^{T}\right)}_{ik}}{u_{ik}^{t}}\geq VQ_{i}V^{T},$$

since

(30) $${\left(U_{i*}VQ_{i}V^{T}\right)}_{ik}=\sum_{a=1}^{K}u_{ia}{\left(VQ_{i}V^{T}\right)}_{ka}\geq u_{ik}{\left(VQ_{i}V^{T}\right)}_{kk}.$$

According to (26) and (28), expand $H_{Huber}\left(v,v_{kj}^{t}\right)\geq F_{kj}\left(v\right)$ is as follows:

(31) $$\frac{{\left(U^{T}R_{j}UV_{*j}+\alpha VL+\beta VG\right)}_{kj}}{v_{kj}^{t}}\geq {\left(U^{T}R_{j}U\right)}_{kk}+{\left(\alpha L\right)}_{jj}+{\left(\beta G\right)}_{jj},$$

since

(32) $${\left(U^{T}R_{j}UV_{*j}\right)}_{kj}=\sum_{b=1}^{K}{\left(U^{T}R_{j}U\right)}_{bk}v_{bj}\geq {\left(U^{T}R_{j}U\right)}_{kk}v_{kj},$$

(33) $${\left(\beta VG\right)}_{kj}=\beta \sum_{b=1}^{N}v_{kb}G_{bb}\geq \beta v_{kj}G_{jj},$$

and

(34) $$\begin{array}{l} {\left(\alpha VD\right)}_{kj}=\alpha \sum_{c=1}^{N}v_{kc}D_{cc}\geq \alpha v_{kj}D_{jj}\\ \;\geq \alpha v_{kj}{\left(D-W\right)}_{jj}=\alpha v_{kj}L_{jj}. \end{array}$$

So, $H_{Huber}\left(u,u_{ik}^{t}\right)\geq F_{ik}\left(u\right)$ and $H_{Huber}\left(v,v_{kj}^{t}\right)\geq F_{kj}\left(v\right)$ can be obtained. In other words, the auxiliary functions F*_ik_* (*u*) and F*_kj_* (*v*) of the updated rules (16) and (17) are non-increasing, and the derivation of theorem 1 is completed. Finally, the convergence of the loss function *O* *_Huber-SGNMF_* is proved.

**Figure 2:**
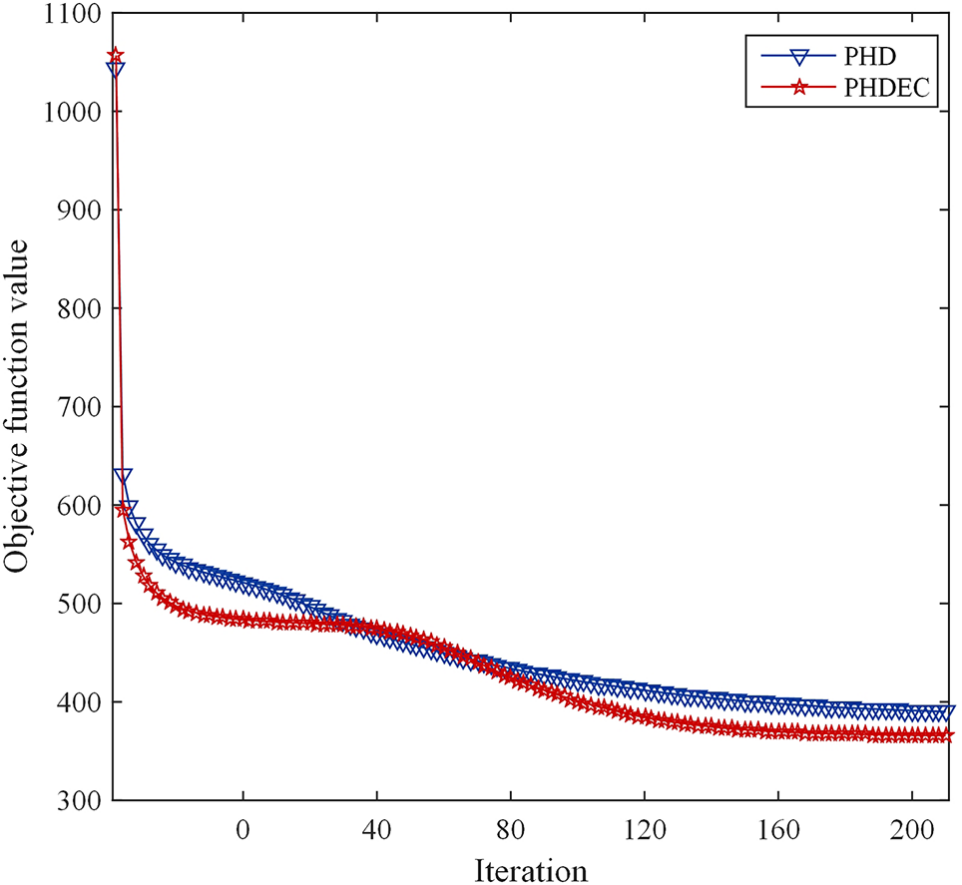
Convergence analysis curve of Huber-SGNMF model. Each curve represents a dataset. PHD and PHDEC are the datasets used in the experiment.

The corresponding convergence analysis curve is shown in **Figure 2**.

## Results and Discussion

### Datasets

Five gene expression datasets downloaded from TCGA are used in the experiment. TCGA is a gene data sharing system that contains information on thousands of cancer patients and has made great contributions to the path of human exploration of cancer genomics. The experiment used five datasets including cholangiocarcinoma (CHOL), colon adenocarcinoma (COAD), head and neck squamous cell carcinoma (HNSC), pancreatic cancer (PAAD), and esophageal cancer (ESCA).

To explore the association between genes and multiple cancers, diseased samples from multiple datasets are integrated into one dataset. In detail, the detesteds PAAD, HNSC, and COAD are integrated into one dataset, which is represented as PHD. The detesteds PAAD, HNSC, and COAD are integrated into one dataset, which is represented as PHD. These two integrated datasets contain only diseased samples of different diseases. Datasets are standardized before using, and the data normalization scales data to specific time intervals. Pre-processing data speeds up searching for the best solution and optimizes convergence speed. Since high-dimensional gene expression data contains a large amount of redundant information, PCA (Wu et al., 2017) is used to reduce the dimensions to 2,000 genes in the pre-processing.

### Model Robustness

**Figure 3:**
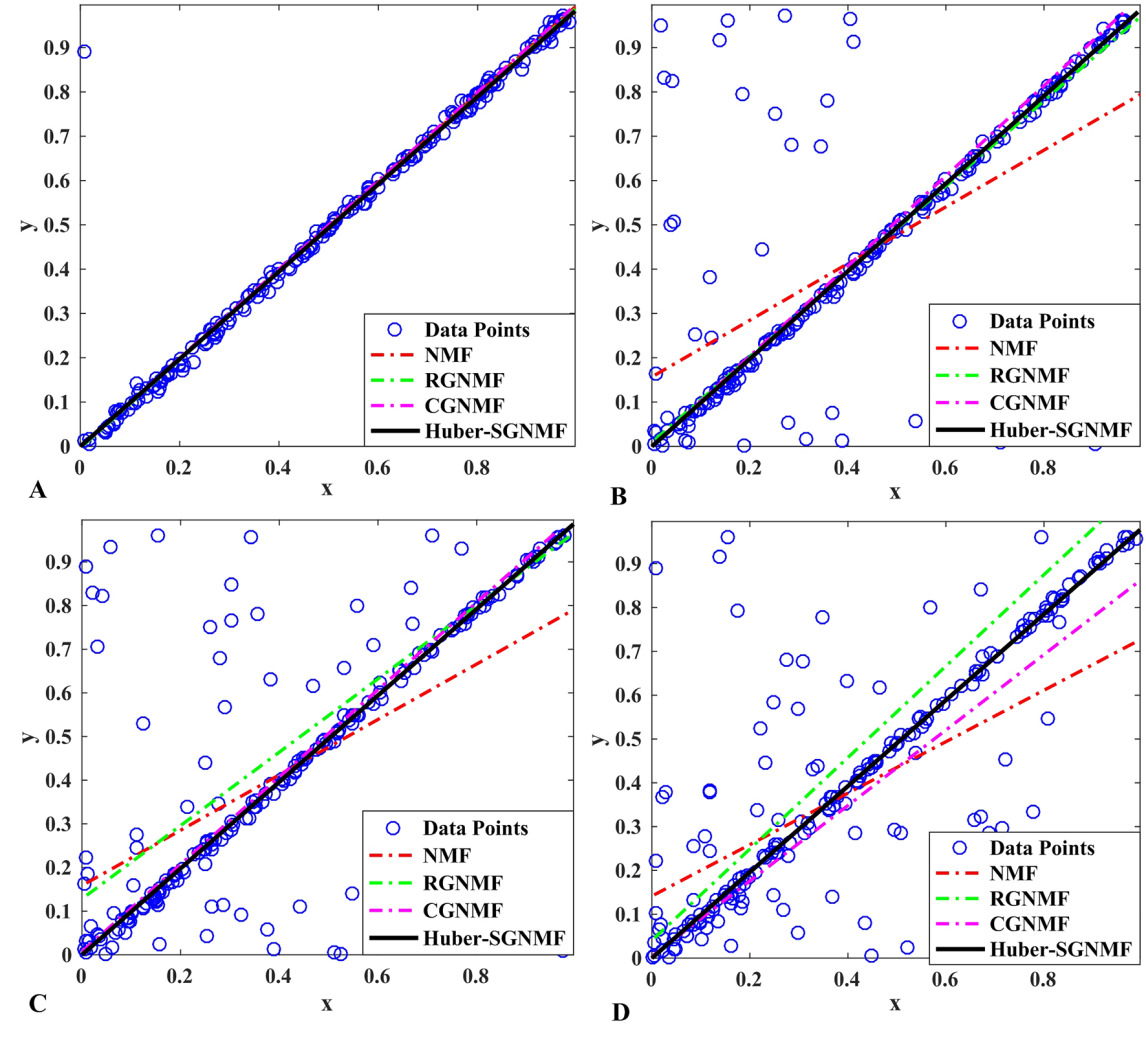
In the case of different data points are contaminated, NMF, RGNMF, CGNMF, and Huber-SGNMF restore 200 synthetic two-dimensional data points: **(A)** the data contains 1 noise or outlier, **(B)** the data contains 50 noise or outliers, **(C)** the data contains 100 noise or outliers, **(D)** the data contains 150 noise or outliers.

To analyze the robustness of RGNMF, CGNMF, and Huber-SGNMF, we apply these methods to a composite dataset consisting of 200 two-dimensional data points (**Figure 3A**). All data points are distributed in one dimensional space. In **Figure 3A**, there is only one contaminated point, and each model can restore the original data normally. The contaminated points in **Figures 3B–D** are 50 points, 100 points, and 150 points, respectively. In the case where a part of the data is contaminated, only Huber-SGNMF successfully restores the original data. CGNMF and RGNMF are affected by some noise or outliers when restoring data, while NMF is most affected by noise or outliers.

### Parameter Selection

**Figure 4:**
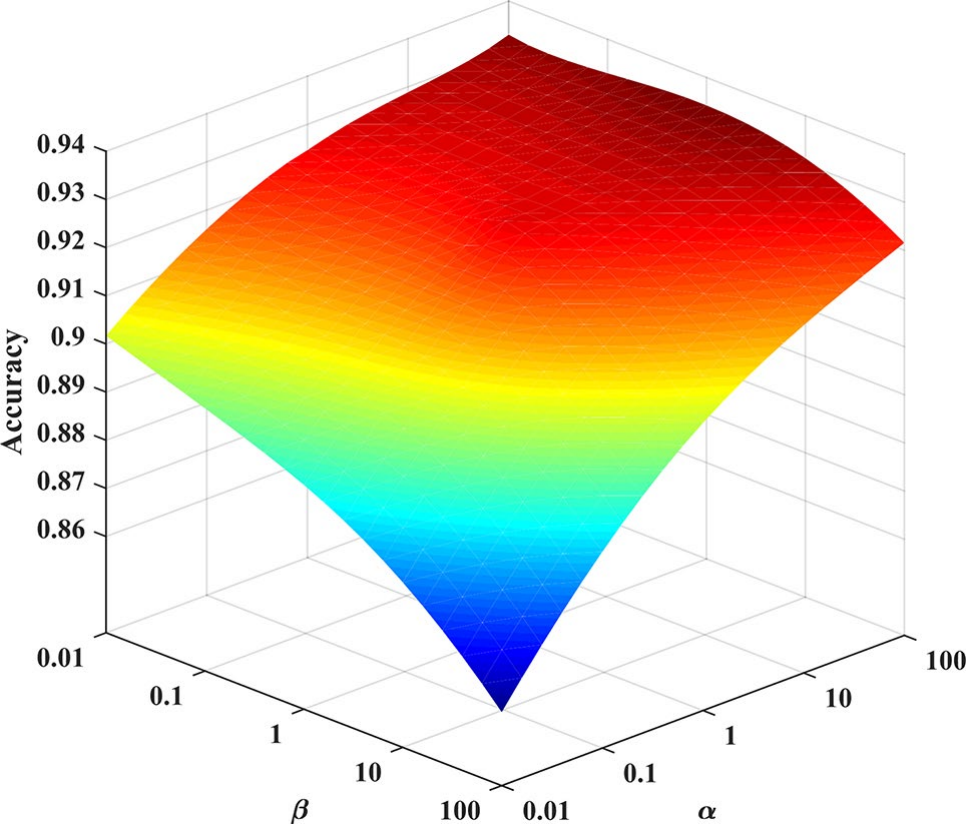
Optimal parameter selection for the Huber-SGNMF model on the PHD dataset. Huber-SGNMF is set with parameters *α* = 100 and *β* = 0.01.

In the experiment, we consider the effect of each parameter on the solution model. A grid search method is used to find the optimal parameters of the model. The grid search range is [10^-2^∼10^2^]. As shown in **Figure 4**, the PHD dataset is used as an example to find the optimal parameters of the Huber-SGNMF model. Specifically, the two datasets are set to the same parameters *α* = 100 and *β* = 0.01 Other methods in the experiment are set up with prior parameters or grid searches to find the optimal parameters.

### Performance Evaluation and Comparisons

To prove the validity of the performance of the model, six states of the art methods including RPCA (Liu et al., 2013), NMF (Lee and Seung, 1999), SNMF (Kim and Park, 2007), GNMF (Cai et al., 2011), RGNMF (Wang et al., 2016a), CGNMF (Mao et al., 2014), and Huber-NMF (Du et al., 2012) are compared with Huber-SGNMF. In the experiment, the basic matrix and the coefficient matrix are used to differentially gene selection and cluster analysis, respectively.

#### Feature Selection Results and Analysis

Feature selection is the selection of representative features from multiple feature values (Yu and Liu, 2003). In the analysis of cancer data, the feature selection is to find differentially expressed genes for cancer (that is, pathogenic genes). This is of great significance in exploring the link between cancer and genes (Chen et al., 2017). For each method, the top 500 genes with the greatest differential expression are analyzed.

The GeneCards (https://www.genecards.org/) system is used to download all gene libraries associated with the disease. The selected genes are compared with the gene bank to select overlapping genes and obtain a corresponding relevance score. The relevance score is the indicator that GeneCards assesses the association between the gene and the disease. The higher the relevance score is, the greater the intimacy of the gene and the disease. The average relevance score (ARS) and the maximum relevance score (MRS) are used to evaluate the performance of the model.

The specific experimental results of the seven methods are listed in **Table 1**. The results show that the genes selected by Huber-SGNMF model have higher ARS and MRS. This means that the model can effectively find the genes associated with cancer. **Table 2** lists the genes for the top 10 largest relevance scores selected by the Huber-SGNMF model on the PHD dataset. The detailed genetic analysis is as follows. 

**Table 1:** Relevance scores for seven methods.

	NMF	SNMF	GNMF	RGNMF	RPCA	CGNMF	Huber-NMF	Huber-SGNMF	
PHD	MRS	116.4	113.99	116.4	164.03	**194.01**	113.99	164.03	164.03
	ARS	22.64	21.75	22.03	22.16	26.03	21.75	25.56	**27.19**
PHDEC	MRS	92.51	96.36	153.66	124.37	**172.9**	164.91	145	**172.9**
	ARS	29.18	30.05	36.58	27.87	37.83	35.07	35.93	**44.97**

Bolded texts denoted best experimental results.

**Table 2:** Detailed analysis of the differentially expressed genes in PHD dataset.

Gene name	Relevance score	Gene official name	Related diseases
CTNNB1	164.03	Catenin beta 1	Colorectal cancer and pilomatrixoma
ERBB2	152.33	Erb-B2 receptor tyrosine kinase 2	Lung cancer and ovary adenocarcinoma
CDH1	149.92	Cadherin 1	Gastric cancer and breast cancer
TGFBR2	102.74	Transforming growth factor beta receptor 2	Colorectal cancer and esophageal cancer
CDK4	93.35	Cyclin dependent kinase 4	Myeloma and melanoma
EPCAM	86.79	Epithelial cell adhesion molecule	Pancreatic cancer and gastrointestinal carcinoma
GNAS	76.17	GNAS complex locus	Osseous heteroplasia
ERBB3	74.35	Erb-B2 receptor tyrosine kinase 3	Transitional cell carcinoma
CEACAM5	59.9	Carcinoembryonic antigen related cell Adhesion molecule 5	Colorectal cancer and lung cancer
MAP2K2	51.51	Mitogen-activated protein kinase kinase 2	Head and neck squamous cell carcinoma

CTNNB1 is a protein-coding gene from which the protein encoded by the gene forms part of an adhesion-linked protein complex. Mutations in the CTNNB1 proto-oncogene are associated with most human colorectal epithelial tumors, and a significant increase in expression in the same tumor may indirectly or directly lead to intestinal adenocarcinoma (Wang et al., 2011). Moreover, deep sequencing of patients with pancreatic ductal adenocarcinoma also found CTNNB1 mutations (Honda et al., 2013; Javadinia et al., 2019). Multiple studies have shown that CTNNB1 mutation analysis is important for PAAD and COAD (Kubota et al., 2015).

ERBB2, commonly referred to as HER2, may be critical for enhancing the synergistic effect of PI3K inhibitors in HNSC patients (Michmerhuizen et al., 2019). It is generally believed that dysregulated ERBB2 signaling plays a key role in the development of pancreatic cancer (Lin et al., 2019). For the treatment of intestinal adenocarcinoma, ERBB2 mutations and amplification in small intestinal adenocarcinoma patients also make a great contribution (Adam et al., 2019). Recent studies have shown that HER2 targeted therapy has significantly improved outcomes in patients with breast and stomach problems with ERBB2 mutation/amplification (Meric-Bernstam et al., 2019).

The CDH1 gene plays a regulatory role in cell growth (Nagai et al., 2018), and the CDH1 gene located on chromosome 16q22.1 is considered to be a tumor suppressor of diffuse gastric cancer. By measuring the methylation profile of gastric cancer and breast cancer patients, it is found that CDH1 is closely related to low protein expression (Wang et al., 2016b; Wang et al., 2016c). Studies have shown that abnormal expression of CDH1 gene leads to uncontrolled growth of tumor cells (Dial et al., 2007; Chen et al., 2012).

The above experimental results show that Huber-SGNMF model can find pathogenic genes more effectively. Although some genes have not been confirmed, they may be a key part of solving cancer problems in the future.

#### Clustering Results and Analysis

After the Huber-SGNMF model reduces the dimensions of the data, the coefficient matrix is used for k-means clustering. Sample clustering is a common analytical method for cancer diagnosis and molecular subtype discrimination (Xu et al., 2019). Moreover, multiple evaluation criteria accuracy (ACC), recall (R), precision (P), and F-measure (F) are adopted to judge the model to be feasible and effective. ACC is an evaluation standard that can visually reflect the clustering of samples. It is defined as follows:

(35) $$ACC=\frac{\sum_{i=1}^{n}\delta \left(a_{i},\text{map}\left(b_{i}\right)\right)}{n},$$

Where *δ* (•) and map (•) represent function permutation and delta mapping function, respectively. The actual sample label, the predicted sample label, and the total number of samples are denoted by *a, b* and *n*, respectively.

Considering clustering accuracy alone does not fully demonstrate clustering performance, and more evaluation criteria need to be introduced. The clustering results can be divided into true positive (TP), true negative (TN), false positive (FP) cases, and false negative (FN) according to real and predictive labels. These four measures are listed in **Table 3**. The detailed evaluation criteria are as follows.

**Table 3:** Clustering result confusion matrix.

The true situation	Clustering result	
	Positive	Negative
Positive	TP (true positive)	FN (false negative)
Negative	FP (false positive)	TN (true negative)

(36) $$R=\frac{TP}{TP+FN},$$

(37) $$P=\frac{TP}{TP+FP},$$

(38) $$F=\frac{2\times R\times P}{R+P}.$$

Since *R, P,* and *F* can only reflect the clustering performance of a certain sample categories, for multi-category problems, the average of each category of indicators is usually used as the evaluation criterions:

(39) $$Macro-R=\frac{1}{n}\sum_{i=1}^{n}R_{i},$$

(40) $$Macro-P=\frac{1}{n}\sum_{i=1}^{n}P_{i},$$

(41) $$Macro-F=\frac{2\times Macro-R\times Macro-P}{Macro-R+Macro-P},$$

where *n* represents the number of sample categories.

According to the above evaluation criterions, each algorithm is performed 50 times to get an average result. Since the initialization matrix is random, the average value can reduce the chance of the algorithm. **Table 4** lists the comparative experiments of seven methods based on four evaluation criterions. Compared with the other six methods, our proposed model has the excellent clustering performance under the four evaluation criterions. The specific analysis of the clustering results is as follows:

1. Since the squared loss of the original NMF is sensitive to noise and outliers, the squared loss is replaced by Huber loss to improve the robustness of the algorithm. The experimental results show that the clustering performance of RPCA, CGNMF, RGNMF, Huber-NMF, and Huber-SGNMF is higher than standard NMF and GNMF. The reason is that both NMF and GNMF use square loss while other methods use more robust loss function. Moreover, the experimental results show that the robustness of the Huber loss model is higher than the *L*_2,1_ -norm loss and correntropy loss. The RPCA model has higher performance as a state-of-the-art algorithm and is still lower than Huber-SGNMF. The Huber loss use *L*_1_ -norm or *L*_2_ -norm to different data, which can effectively reduce the influence of noise and outliers and enhance the robustness of the algorithm. Compared with NMF, the clustering accuracy of Huber-SGNMF model on the two datasets increased by 4.90 and 5.68%, respectively.
2. Assuming that data points are related in a high-dimensional state, they should also be relevant in low-dimensional representations. However, the association between data points is difficult to preserve when the data is mapped to low-dimensions. The manifold structure preserves the spatial structure of high-dimensional data in low-dimensional representations, enhancing the correlation between data points. Constructing a sample association graph of gene expression data to preserve the relationship between the samples. The experimental results of several models (NMF and GNMF, Huber-NMF, and Huber-SGNMF) show that the clustering performance of the model with the addition of graph regularity constraints is improved. Compared with Huber-NMF, Huber-SGNMF has improved clustering accuracy by 1.73 and 2.99% in the two datasets, respectively.
3. Matrix sparseness removes redundant data and simplifies model calculations. The sparsity constraint of the coefficient matrix removes redundant features and improves clustering performance. The experimental results of SNMF and Huber-SGNMF prove this. Compared with SNMF, since Huber-SGNMF improves the loss function and manifold structure, the clustering accuracy in the two datasets is increased by 1.35 and 4.02%, respectively.

**Table 4:** Clustering effect for seven methods.

Dataset	Evaluation	NMF	SNMF	GNMF	RGNMF	RPCA	CGNMF	Huber-NMF	Huber-SGNMF
PHD	ACC (%)	85.38 ± 1.24	88.93 ± 0.58	86.05 ± 1.97	86.50 ± 1.84	86.37 ± 2.04	87.18 ± 1.43	88.55 ± 0.98	**90.36 ± 0.91**
	Macro-R (%)	82.99 ± 1.57	86.86 ± 0.82	81.02 ± 1.09	84.28 ± 2.40	84.10 ± 2.79	85.00 ± 1.79	86.41 ± 1.27	**88.50 ± 1.19**
	Macro-P (%)	84.88 ± 1.74	89.08 ± 0.86	83.55 ± 3.76	85.68 ± 2.74	85.58 ± 2.96	86.77 ± 2.02	88.32 ± 1.36	**90.18 ± 1.28**
	Macro-F (%)	83.92 ± 1.65	87.95 ± 0.84	82.25 ± 3.51	84.92 ± 2.60	84.83 ± 2.88	85.87 ± 1.90	87.35 ± 1.31	**89.33 ± 1.23**
PHDEC	ACC (%)	69.84 ± 0.26	71.51 ± 0.31	70.15 ± 0.08	71.86 ± 0.69	75.02 ± 0.32	73.81 ± 0.27	72.53 ± 0.21	**75.52 ± 0.20**
	Macro-R (%)	63.95 ± 0.18	65.33 ± 0.14	61.98 ± 0.38	64.45 ± 0.87	68.37 ± 0.28	66.74 ± 0.15	67.09 ± 0.07	**69.02 ± 0.07**
	Macro-P (%)	61.34 ± 0.26	62.45 ± 0.19	58.77 ± 0.10	62.80 ± 0.97	**65.81 ± 0.50**	64.47 ± 0.27	63.92 ± 0.25	65.56 ± 0.25
	Macro-F (%)	64.17 ± 0.20	63.79 ± 0.21	60.24 ± 0.27	63.49 ± 0.87	66.92 ± 0.29	65.51 ± 0.17	65.34 ± 0.12	**67.17 ± 0.10**

Bolded texts denoted best experimental results.

In summary, the experimental results based on the four evaluation indicators demonstrate the excellent clustering performance of the Huber-SGNMF model. Compared with NMF, the clustering performance of Huber-SGNMF has improved 5.30 and 4.49% on average in PHD dataset and PHDEC dataset, respectively. Huber-SGNMF clustering performance improves 1.93 and 2.07% on average compared to Huber-NMF. The above experimental results strongly prove the effectiveness of Huber-SGNMF in clustering performance.

## Conclusion

In this paper, we propose a novel model based on Huber loss: Huber-SGNMF, which is dedicated to samples clustering and differentially expressed gene selection. On the one hand, the squared loss is replaced by Huber loss to enhance algorithm robustness. On the other hand, sparse penalty and graph regularization terms are added to the model to enhance the sparsity of the matrix and preserve data geometry information. Numerous experimental results confirm that the Huber-SGNMF method is more effective. In the future work, we will actively explore more effective constraints based on the traditional NMF method to improve the robustness and sparsity of the method.

## Data Availability Statement

The datasets for this study can be downloaded in the The Cancer Genome Atlas [https://cancergenome.nih.gov/]. 

## Author Contributions

C-YW and NY proposed and designed the algorithm. J-XL demonstrated the robustness of the algorithm and analyzed the experimental data. C-YW and C-HZ drafted the manuscript. 

## Conflict of Interest

The authors declare that the research was conducted in the absence of any commercial or financial relationships that could be construed as a potential conflict of interest.
